# Causal Effects of Inflammatory Bowel Disease on Lung Function and Disease: A Two‐Sample Mendelian Randomization Study

**DOI:** 10.1002/hsr2.71107

**Published:** 2025-07-27

**Authors:** Xuewei Fu, Hao Wu, Chao Deng, Qifeng Ou, Yiyuan Yang, Yuezhan Li

**Affiliations:** ^1^ Department of Emergency, Hainan General Hospital Hainan Affiliated Hospital of Hainan Medical University Haikou China; ^2^ Department of Gastroenterology, Xiangya Hospital Central South University Changsha China; ^3^ National Clinical Research Center for Geriatric Disorders, Xiangya Hospital Central South University Changsha Hunan; ^4^ Department of Plastic and Cosmetic Surgery, Xiangya Hospital Central South University Changsha China; ^5^ Regenerative Medicine Institute, School of Medicine National University of Ireland (NUI) Galway Ireland; ^6^ Department of Dermatology, Changhai Hospital Naval Medical University Shanghai China

**Keywords:** asthma, Crohn's disease, lung disease, lung function, Mendelian randomization, ulcerative colitis

## Abstract

**Background and Aims:**

The increasing prevalence of Crohn's disease (CD) and ulcerative colitis (UC)—the two main forms of inflammatory bowel disease (IBD)—has made these conditions a growing concern in global public health. While IBD primarily impacts the gastrointestinal tract, emerging evidence suggests its extraintestinal effects, especially on the respiratory system. The correlation between the lungs and the gastrointestinal system has drawn attention to the potential pulmonary complications in IBD patients. The causal link between IBD and lung function or related disease remains inconclusive.

**Methods:**

Leveraging publicly available genome‐wide association study (GWAS) data sets, we performed a two‐sample Mendelian randomization analysis to investigate potential links between ulcerative colitis, Crohn's disease, and various aspects of lung function or respiratory illness. Through meticulous quality control measures, we pinpointed SNPs with robust associations to UC and CD. The inverse‐variance weighted method was our primary analytic approach. Additionally, we evaluated heterogeneity, explored potential pleiotropy, and performed sensitivity tests.

**Results:**

We conducted an analysis using GWAS data obtained from the European population, identifying 54 and 77 SNPs for association studies related to lung function and disease in UC and CD, respectively. The results from the MR analysis revealed that neither UC nor CD exhibited a discernible impact on lung function, encompassing metrics such as total lung volume, forced vital capacity, forced expiratory volume in the first second, the forced vital capacity/forced expiratory volume in the first second ratio, and peak expiratory flow. Additionally, neither UC nor CD demonstrated an elevated risk of idiopathic pulmonary fibrosis, chronic obstructive pulmonary disease, or pulmonary cancer. In contrast, CD presented a heightened risk of asthma compared to UC.

**Conclusion:**

CD has been associated with a higher risk of developing asthma in the European population.

## 1 Introduction

Crohn's disease (CD) and ulcerative colitis (UC), collectively referred to as inflammatory bowel diseases (IBD), have evolved as pressing global health concerns. The worldwide occurrence of IBD is on the rise, with North America accounting for over 2 million affected individuals, and Europe approximately 3.2 million. The worldwide tally soars into the multi‐millions, highlighting the pervasive nature and crucial implications of IBD [1, 2, 3]. Although IBD primarily targets the gastrointestinal tract, there is a growing awareness of its extraintestinal manifestations, notably in the respiratory system [4, 5]. The lungs and the gastrointestinal system, with shared embryological origins, present an intriguing interconnection. While pulmonary disorders are relatively rare, their incidence may be elevated in subjects with IBD than in the general population. For instance, a prospective study highlighted that IBD patients exhibited notably diminished lung function compared to healthy controls. This decrement in pulmonary function was more pronounced during active disease phases than during remission periods [6]. Another large population‐based cohort study revealed that individuals with IBD experienced elevated incidences of specific respiratory conditions, including asthma and bronchiectasis, when contrasted with the general population [7]. Conversely, some scholars argue that in pediatric and adolescent IBD patients who exhibit no respiratory symptoms, pulmonary involvement is insubstantial. Routine screening for asymptomatic individuals seems unwarranted and is not recommend for these younger demographics [8]. However, these observational studies inevitably face limitations related to confounding bias and reverse causation, leaving the causal link between IBD and lung function or respiratory diseases uncertain.

Mendelian Randomization (MR) is a robust statistical method that leverages genetic polymorphisms, often single nucleotide polymorphisms (SNPs), as proxy variables to examine the cause‐and‐effect relationship between a specific exposure and its corresponding outcome [9]. The core premise of MR is grounded in the principle that genomic variations are randomly allocated during conception and thus are free from confounding variables that often plague observational research. By utilizing these proxy variables, MR can circumvent challenges such as reverse causation or residual confounding, offering a more unbiased assessment of causality. In general, MR is akin to a natural randomized controlled trial, where genetic variants serve as the randomization tool [10].

In this study, we implemented a two‐sample MR design to assess whether there is a potential causal relationship between UC, CD, and lung function as well as respiratory illnesses. We utilized extensive genome‐wide association study (GWAS) summary statistics to perform this analysis. Lung function was evaluated by analyzing indicators such as total lung volume, forced expiratory volume in 1 s (FEV1), forced vital capacity (FVC), and peak expiratory flow (PEF), with data sourced from the UK Biobank. Additionally, we explored how genetic predisposition to UC and CD is related to a spectrum of lung diseases—including chronic obstructive pulmonary disease (COPD), idiopathic pulmonary fibrosis (IPF), asthma, and various forms of lung malignancy—across European‐descent populations. Through this analysis, our objective was to provide an exhaustive insight into the potential cause‐and‐effect relationship between UC, CD and both lung function and associated diseases.

## 2 Methods

### 2.1 GWAS Repositories for Inflammatory Bowel Disease

The data pertaining to IBD, including both UC and CD, were obtained from a comprehensive meta‐analysis that integrated findings from the UK Inflammatory Bowel Disease Genetics Consortium and the International Inflammatory Bowel Disease Genetics Consortium. The aggregated data set consisted of 25,042 individuals diagnosed with IBD and 34,915 control participants. Within this cohort, there were 12,366 UC cases (with 33,609 corresponding controls) and 12,194 CD cases (with 28,072 corresponding controls). Diagnosis of IBD subtypes was based on rigorous clinical criteria, incorporating findings from endoscopy, radiological imaging, and histopathological examination [11]. The primary GWAS summary statistics are publicly accessible via the Wellcome Sanger Institute's database.

### 2.2 Sources of GWAS Data Pertaining to Lung Function and Respiratory Disease Characteristics

We employed summary‐level data obtained from existing GWAS on lung function, specifically sourced from the UK Biobank, comprising a sample size ranging from 32,860 to 321,047 individuals of European ancestry [12, 13]. These GWAS evaluated a range of pulmonary performance metrics, such as FVC, FEV1, FEV1/FVC, PEF, and total lung volume. Spirometric time‐series data were used to obtain measurements of FVC, FEV1, and PEF, while total lung volume was measured using magnetic resonance imaging data from the UK Biobank. For COPD, the GWAS data incorporated 8631 cases (never smoke) and 120,544 controls of European ancestry [14]. COPD was identified using pre‐bronchodilator spirometry results, applying adapted Global Initiative for Chronic Obstructive Lung Disease (GOLD) guidelines to define cases exhibiting moderate airflow restriction. Regarding asthma, we utilized data from the NHGRI‐EBI GWAS Catalog (GCST90014325) [15], consisting of 56,167 cases and 352,255 controls. Asthma criteria were established according to the International Classification of Diseases (ICD)‐10 code J45. For IPF, the data from the FinnGen consortium R9 incorporated 2,018 cases and 373,064 controls. The criteria of IPF were based on the ICD‐10 code J84. For lung cancer, our source was a meta‐analysis that included 29,266 individuals with cases and 56,450 controls of European descent. This analysis primarily identified histological subtypes of lung cancer, with adenocarcinoma accounting for 11,273 cases, followed by squamous cell carcinoma with 7426 cases and small cell carcinoma with 2664 cases [16].

### 2.3 Genetic Instrumental Variables Selection

The criteria for genetic instrument selection have been described previously [17]. To minimize the risk of weak instrument bias for each SNP, we computed *F* statistics using the formula *F *= (beta/se)^2^. SNPs with *F* values substantially above 10 were considered robust instrumental variables. We further screened and excluded SNPs with direct associations to lung function traits, respiratory diseases, or smoking behavior using the Phenoscanner V2 database (http://www.phenoscanner.medschl.cam.ac.uk/). The removed SNPs are mainly related to asthma, FEV1, FVC, PEF, and tobacco smoking (Supporting Information S1: Table S1). In this study, we ultimately pinpointed 54 index SNPs as proxy variables for UC and 77 index SNPs for CD (Supporting Information S1: Table S2).

### 2.4 Mendelian Randomization Estimates

The primary analysis utilized the random‐effects inverse variance weighted (IVW) approach, along with additional evaluations using MR–Egger and weighted median (WM) techniques [18, 19, 20]. When similar directional effects were observed across the IVW, MR–Egger, and WM estimators, this enhanced the reliability of the inferred causal relationship. To further refine our analysis, we conducted MR Pleiotropy Residual Sum and Outlier tests, enabling the identification and removal of outlier SNPs that contributed disproportionately to heterogeneity in the causal estimates. Heterogeneity among the instrumental variables was quantified using Cochran's *Q* statistic. To assess the presence of horizontal pleiotropy, we utilized the MR–Egger intercept and performed leave‐one‐out (LOO) sensitivity analyses. Furthermore, the Steiger test was performed to confirm the direction of observed causal relationships [21]. Statistical computations were performed in R (version 4.3.1), employing the TwoSampleMR (version 0.5.7) and MRPRESSO (version 1.0) packages. To account for multiple testing, we adopted a conservative strategy by applying a Bonferroni correction, adjusting the significance threshold to 0.0027 (calculated as 0.05 divided by 18) [22]. Associations yielding a *p* value below this adjusted threshold were interpreted as strong evidence for causality, while those with *p* values ranging from 0.0027 to 0.05 were considered indicative of a potential association.

## 3 Results

**Figure 1:**
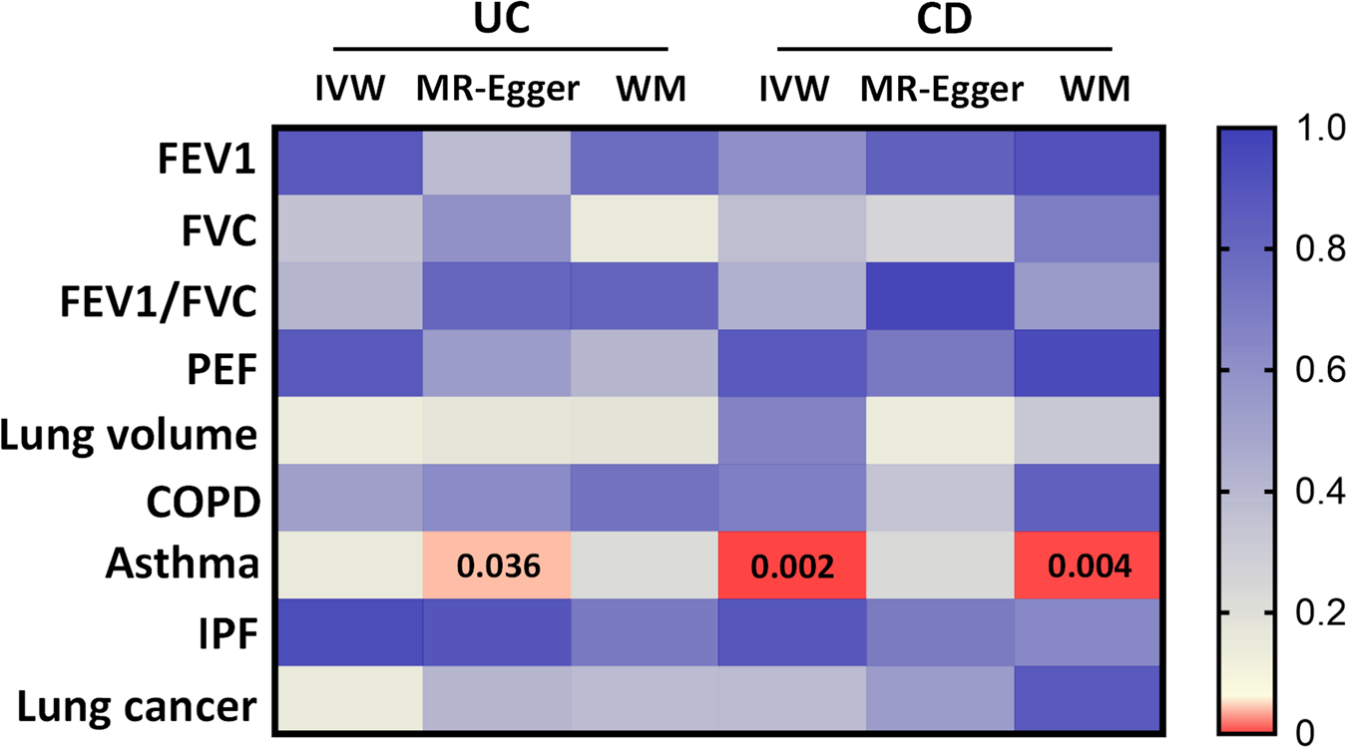
MR analysis of UC and CD impact on lung function or disease. Block colors indicate *p* values for each MR assessment. Significance set at *p* < 0.0027.

The specifics of all GWAS utilized in this study are summarized in Table 1. We selected 54 IVs (with *F* statistics ranging from 30.46 to 194.41) for the genetic prediction of UC and 77 IVs (with *F* statistics ranging from 29.79 to 489.57) for CD. After outlier exclusion using MR‐PRESSO, the main analysis was performed using the random‐effects IVW, supplemented by MR–Egger and WM. The resulting causal effects of UC and CD on lung function measures and respiratory disease traits are visually represented in a heatmap (Figure 1).

**Table 1:** GWAS details for the Mendelian randomization analysis.

Trait/Phenotype	Resources	Participants
Ulcerative colitis	UKIBDGC + IIBDGC	12,366 European ancestry cases
		33,609 European ancestry controls
Crohn's disease	UKIBDGC + IIBDGC	12,194 European ancestry cases
		28,072 European ancestry controls
FEV1	PMID: 30804560	321,047 European ancestry individuals
FVC	PMID: 30804560	321,047 European ancestry individuals
FEV1/FVC	PMID: 30804560	321,047 European ancestry individuals
PEF	PMID: 30804560	321,047 European ancestry individuals
Lung volume	PMID: 34128465	32,860 European ancestry individuals
COPD	PMID: 33106845	8631 European ancestry cases
		120,544 European ancestry controls
Asthma	PMID: 34103634	56,167 European ancestry cases
		352,255 European ancestry controls
IPF	FinnGen consortium R9	2018 European ancestry cases
		373,064 European ancestry controls
Lung cancer	PMID: 28604730	29,266 European ancestry cases
		56,450 European ancestry controls

**Figure 2:**
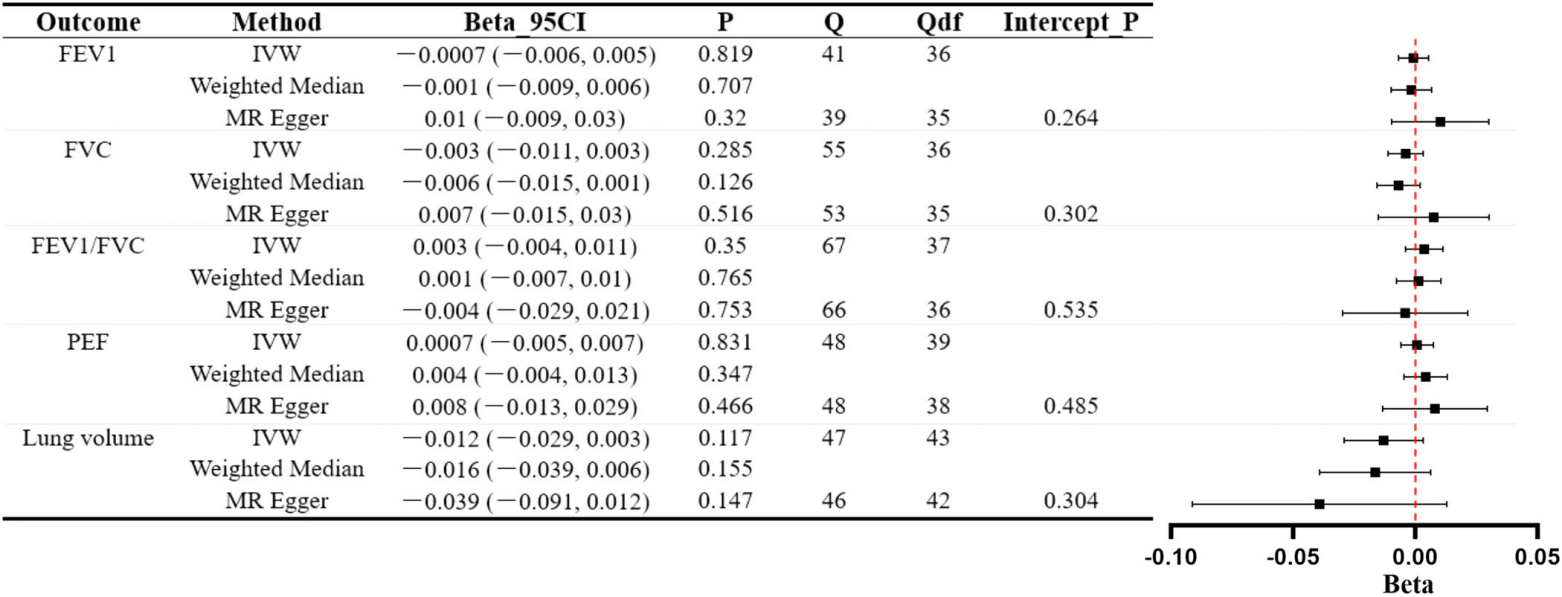
MR analysis of UC's influence on lung function. The forest plot displays three estimates for each outcome (IVW, MR–Egger, weighted median) with accompanying Cochran's Q statistic (Q), degrees of freedom (Qdf), and the *p* value for the MR–Egger intercept (Intercept_P).

In general, the findings from Figure 2 do not sufficiently support the existence of a causal association between UC and various lung function parameters. Scatter plots of MR estimates are shown in Supporting Information S1: Figure S1.

**Figure 3:**
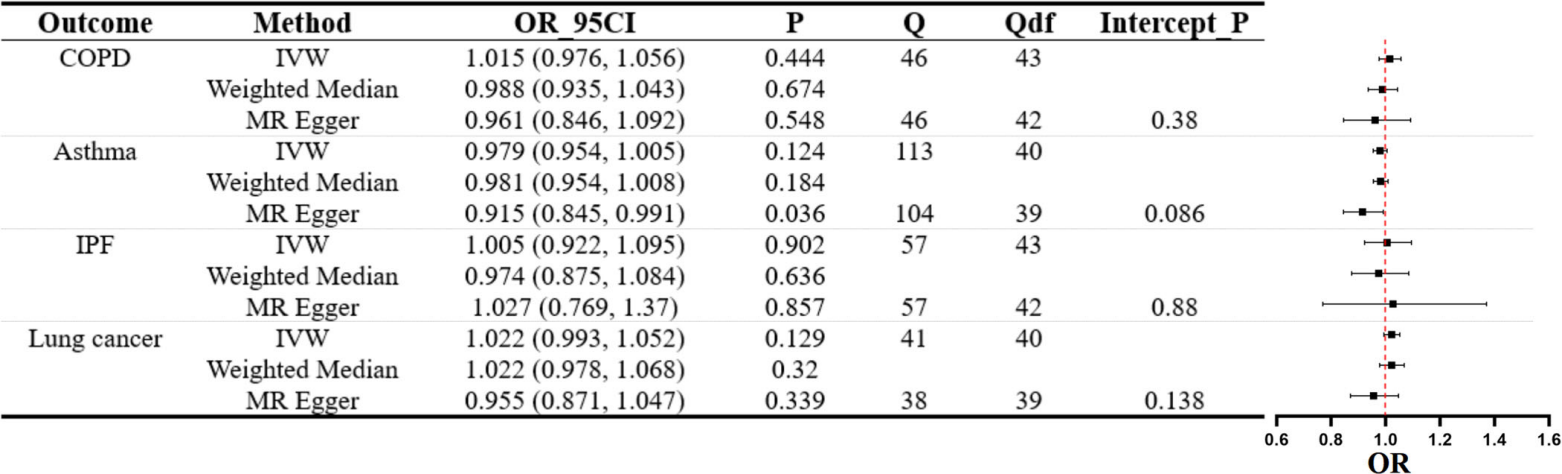
MR assessment of UC's effect on pulmonary diseases. Displayed are forest plots for each outcome using IVW, MR–Egger, and weighted median methods with accompanying Cochran's *Q* statistic (*Q*), degrees of freedom (Qdf), and *p* value for the MR–Egger intercept (Intercept_P).

Next, we delved into the relationship between UC and various pulmonary diseases. Figure 3 findings indicate a lack of substantial evidence to confirm a causal link between UC and several pulmonary diseases. In the results, we observed that although MR–Egger predicted a potential causal relationship between UC and Asthma (MR–Egger OR = 0.915, 95% CI: 0.845–0.991, *p* = 0.036), it is important to consider that our primary methodology was IVW. Moreover, given the propensity of MR–Egger methods to overstate Type I error rates [19], we reassessed the data after applying a Bonferroni correction. Consequently, we conclude that there is insufficient evidence to support a causal link between UC and Asthma. Scatter plots illustrating MR estimates can be viewed in Supporting Information S1: Figure S2.

**Figure 4:**
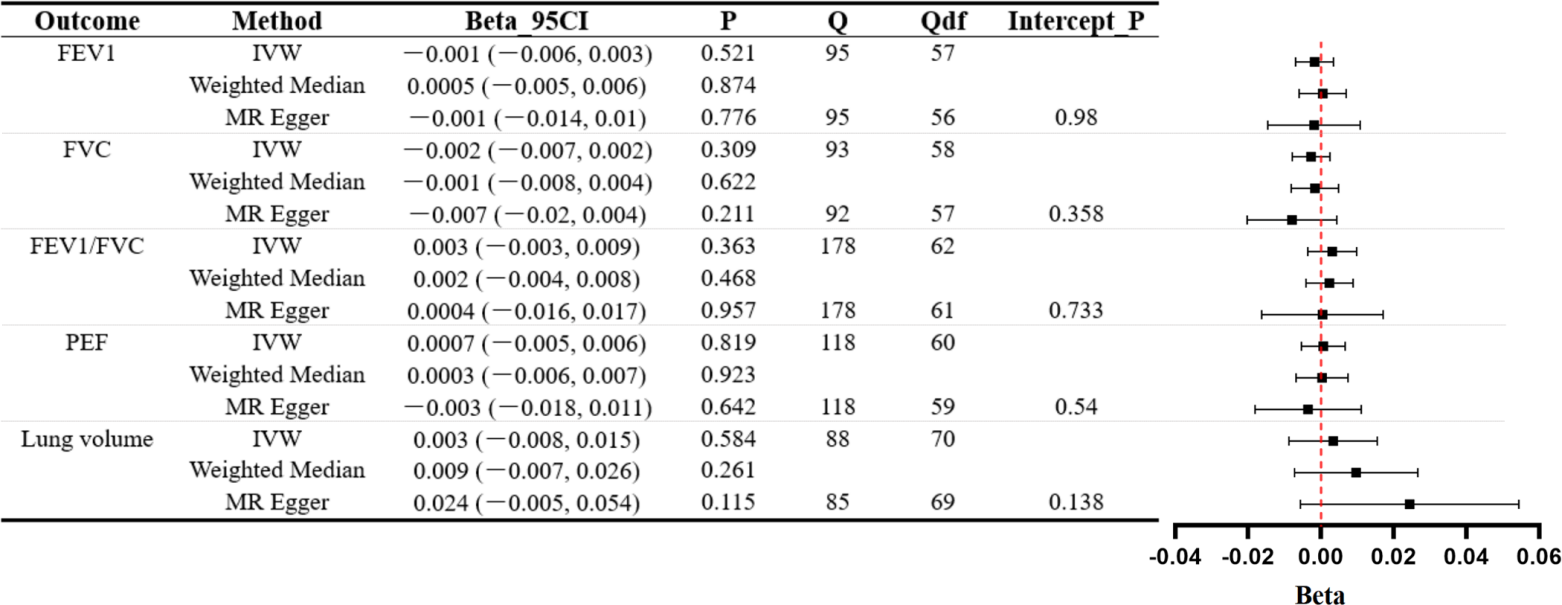
MR evaluation of CD's influence on lung function. Forest plots for each outcome use IVW, MR–Egger, and weighted median methods with accompanying Cochran's Q statistic (Q), degrees of freedom (Qdf), and MR–Egger intercept *p* value (Intercept_P).

Similarly, we replicated the analysis to explore the relationship between CD and both lung function and various lung diseases. Data from Figure 4 suggests that there is no causal association between CD and lung function.

**Figure 5:**
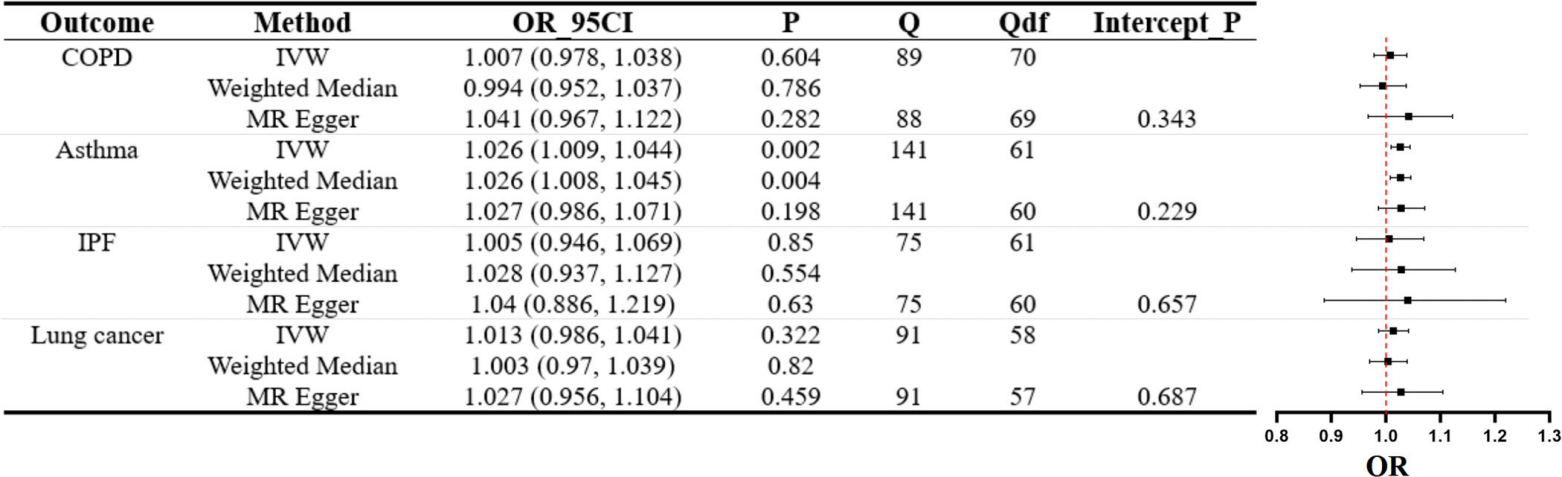
MR evaluation of CD's effect on lung diseases. The forest plot presents three estimates (IVW, MR–Egger, weighted median) for each outcome, accompanied by Cochran's *Q* statistic (*Q*), degrees of freedom (Qdf), and MR–Egger intercept *p* value (Intercept_P).

As described in Figure 5, we observed that CD is associated with an increased risk of asthma. Among these results, our primary method suggests a causal relationship between CD and asthma (IVW OR = 1.026, 95% CI: 1.009–1.044, *p* = 0.002). Supplementary analysis using WM also supports this causal association (WM OR = 1.026, 95% CI: 1.008–1.045, *p* = 0.004). Although the MR–Egger result yields a *p* value greater than 0.05 (MR–Egger OR = 1.027, 95% CI: 0.986–1.071, *p* = 0.198), the consistency in direction among IVW, WM, and MR–Egger methods, coupled with the significant difference in the IVW *p* value following Bonferroni correction (*p *= 0.0022 < 0.027), leads us to conclude that CD is indeed associated with an increased risk of asthma within the European population. However, our findings indicate that CD does not have a causal relationship with COPD, IPF, and lung cancer. Scatter plots of MR estimates are depicted in Supporting Information S1: Figures S3,S4.

Next, we conducted assessments for horizontal pleiotropy and heterogeneity to validate the robustness of our results. The findings from the MR–Egger regression confirmed that there was no horizontal pleiotropy present in the instrumental variables for both UC and CD (all intercept *p* values > 0.05). In addition, we assessed heterogeneity for the IVW and MR–Egger models using Cochran's *Q* statistic. Notable variability in SNP‐specific causal estimates was detected in certain outcomes. However, given the random‐effects model employed by IVW in this study, along with the supplementary assessment using WM, the results of this study remain reliable [23]. Subsequently, we executed a LOO analysis to evaluate the potential influence of individual instrumental variables on outcome measures. Based on the findings (presented in Supporting Information S1: Figures S5,S6), LOO provides significant evidence supporting CD has been associated with an increased risk of asthma within the European population.

## 4 Discussion

IBD impacts not just the gastrointestinal (GI) tract but can also influence other systems. These effects outside the GI tract are commonly referred to as extraintestinal manifestations (EIMs) of IBD [24]. EIMs vary in frequency based on the involved organ and can emerge before or after an IBD diagnosis. They can profoundly affect IBD patients' quality of life (QoL), occasionally more than the intestinal symptoms. Often, EIMs necessitate specialized treatments or consideration when determining intestinal inflammation treatment [25, 26]. Among EIMs, musculoskeletal manifestations are the most prevalent in IBD, impacting up to 46% of patients [27], furthermore, many IBD patients have EIMs related to skin conditions, for instance, cutaneous EIMs occur in 5%–15% of IBD patients [28]. Beyond joints and skin, the eye is another significant site prone to immune‐mediated EIMs. Approximately 2%–7% of IBD patients manifest ocular symptoms. Episcleritis, scleritis, and anterior uveitis as the most frequent ocular EIMs in IBD [29].

Though infrequently observed, pulmonary manifestations in IBD are gaining increased recognition. These pulmonary changes are often underappreciated, particularly when respiratory symptoms precede the IBD diagnosis. The initial identification of a link between respiratory conditions and IBD can be credited to Kraft, who in 1976 described a series of IBD patients with unexplained bronchial suppuration [30]. Subsequent studies have reinforced the link between IBD and pulmonary disorders; however, the exact prevalence of pulmonary involvement in IBD remains elusive and appears inconsistent. Some studies reported minimal respiratory complications, with one observing none in 624 patients [31], and another identifying only 3 out of 1400 IBD cases (0.4%) with respiratory involvement [32]. Establishing a direct correlation between IBD and respiratory disorders becomes challenging in patients who had pre‐existing lung conditions at the time of IBD diagnosis or those who smoke.

To comprehensively investigate the causal link between IBD and lung function as well as pulmonary diseases, we embarked on a Mendelian randomization study. Leveraging one of the most extensive GWAS databases available for UC and CD, our study was designed with an aim to provide a comprehensive and authoritative exploration of this causative association. Initially, we delved into the relationship between UC and CD with critical lung function metrics, including FEV1, FVC, FEV1/FVC, PEF, and lung volume. Anomalies in lung function often signify the presence of underlying pulmonary conditions or diseases; however, in our preliminary analysis, we found no evidence of a causal relationship between UC or CD and pulmonary function indicators. Subsequently, we selected four common pulmonary diseases for further study. As for COPD, a prior study indicated 20% IBD patients presented either an obstructive or restrictive ventilatory defect, a significantly higher proportion than the 5% observed in the control group [33]. However, an updated study indicated that individuals with IBD were significantly more likely to have COPD before the onset of IBD, and that the risk of COPD notably increased post‐IBD diagnosis [34]. In our research, we discovered that certain SNPs have the potential to increase the risk for both IBD and COPD concurrently. When these specific SNPs were excluded from our analysis, our results suggested that there is no cause‐and‐effect relationship between the incidences of IBD and COPD. This emphasizes the significance of considering genetic profiles in understanding the relationship between different diseases, particularly those like IBD and COPD, which may share overlapping genetic risk factors. Likewise, our results indicate no evidence for a causal link between IBD and either IPF or lung cancer. Regarding asthma, prior systematic reviews and meta‐analyses have explored the relationship between asthma and IBD. This comprehensive analysis found a significant link between asthma and both CD and UC, suggesting a need for additional research to understand whether one condition affects the likelihood of developing the other, or if their co‐occurrences arise from common environmental, microbial, and genetic risk factors [35]. Another study conducted in Taiwan investigated the likelihood of asthma developing in adult patients with IBD. This retrospective cohort study included patients with newly diagnosed IBD and compared them with a control group without IBD. The results indicated that patients with IBD faced a 1.50 times higher risk of developing asthma [36]. In our study, only CD was linked to an increased risk of asthma within the European population.

The relationship between CD and asthma is multifaceted, involving complex interactions among immunological, genetic, and environmental factors. CD, a type of IBD, is marked by persistent inflammation that can affect any part of the gastrointestinal tract and is driven by a dysregulated immune response. Asthma is a respiratory disorder characterized by ongoing inflammation of the airways and an increased sensitivity of the bronchial tree. Emerging evidence suggests that individuals with CD may have a higher risk of developing asthma, possibly due to shared genetic susceptibilities, common environmental triggers, and overlapping inflammatory pathways. Furthermore, both conditions are thought to involve disruptions in mucosal barriers and altered microbiomes, which may contribute to their coexistence and interplay. Despite ongoing research, the precise mechanisms underlying the link between CD and asthma remain to be fully elucidated. Both CD and asthma are underpinned by dysregulated immune responses, albeit in different ways. In CD, there is a predominant activation of the Th1/Th17 pathways, while asthma is often associated with a Th2‐dominated response. However, emerging evidence suggests overlapping immune mechanisms. For example, cytokines like interleukin‐13 (IL‐13), which are crucial in asthma pathogenesis, have also been implicated in CD [37]. Environmental factors, particularly those affecting the gut and lung microbiomes, are believed to influence both CD and asthma, the idea of the “gut‐lung axis” has frequently been highlighted [38]. The gut–lung axis describes the two‐way interactions between the respiratory and gastrointestinal systems, mediated by immune mechanisms, microbial compositions, and metabolic pathways. The microbiota in both the lungs and gut is central to this crosstalk. Dysbiosis, an imbalance in this microbiota, has been identified in conditions such as CD and is believed to influence the pathogenesis of respiratory disorders like asthma [39]. Moreover, CD can influence gut permeability, potentially leading to systemic inflammation that may subsequently impact the lungs [40]. Other factors like antibiotic use, dietary habits, and exposure to pollutants have been linked to the development of both conditions, potentially through alterations in the microbiome and subsequent immune responses [41, 42, 43]. In addition, several studies have identified genetic linkages between CD and asthma. For instance, a variant in the ORMDL3 gene is linked to a higher risk for both diseases [44, 45, 46]. This suggests shared genetic susceptibility pathways, potentially contributing to the development of both conditions. Meanwhile, epidemiological research has also shown results regarding the comorbidity of CD and asthma. A prior investigation revealed that the prevalence of asthma within this group of hospitalized patients with IBD was 8%. This prevalence is noteworthy as it highlights the coexistence of asthma in a significant proportion of IBD patients, particularly those with CD [47]. For clinicians, recognizing the possibility of coexisting asthma and CD is essential for optimizing patient care and management. The presence of asthma in individuals with CD may necessitate modifications to standard therapeutic strategies, taking into account the persistent systemic inflammation characteristic of CD as well as the potential for interactions between medications used to treat both conditions. An integrated, multidisciplinary approach may be beneficial, ensuring that treatment plans address both gastrointestinal and respiratory symptoms while minimizing adverse effects. Furthermore, clinicians should remain vigilant for overlapping symptoms and be proactive in screening for comorbid conditions, as early identification and tailored management can improve overall outcomes and quality of life for these patients.

To our knowledge, this is the inaugural study leveraging MR analysis and extensive GWAS data to explore the cause‐and‐effect link between IBD and lung function/disease. Our findings collectively indicate a notable link between CD and a higher risk of developing asthma, suggesting potential overlapping pathophysiological mechanisms or shared risk factors that warrant further investigation. The co‐occurrence of these conditions highlights the importance of comprehensive care and surveillance for respiratory complications in patients with CD. Although our research provides important insights, it is important to recognize its limitations. Our research focuses exclusively on European populations, potentially constraining its global applicability. Furthermore, we did not categorize our findings by gender or age, which could impact the interpretation of results. Additionally, although we leveraged a substantial GWAS sample, we recognize that future data updates might lead to different conclusions. We are committed to remaining vigilant and are prepared to refine our findings with fresh data as it becomes available.

## 5 Conclusion

In conclusion, we found CD has been associated with a higher risk of developing asthma in the European population, but more research is needed to confirm this.

## Author Contributions

**Xuewei Fu:** conceptualization, data curation, methodology, formal analysis, investigation, software, writing – original draft, visualization. **Hao Wu:** formal analysis, investigation, software. **Chao Deng:** software, visualization. **Qifeng Ou:** data curation, formal analysis, investigation. **Yiyuan Yang:** conceptualization, project administration, supervision, writing – review and editing. **Yuezhan Li:** conceptualization, supervision, writing – review and editing.

## Ethics Statement

The Medical Ethics Committee of Hainan General Hospital reviewed this study and granted a formal waiver for ethical approval (No. Med‐Eth‐Re[2025] 353), as the research involved only retrospective analysis of anonymized data.

## Consent

The Medical Ethics Committee of Hainan General Hospital granted a formal waiver for informed consent.

## Conflicts of Interest

The authors declare no conflicts of interest.

## Transparency Statement

The corresponding authors, Yiyuan Yang and Yuezhan Li, affirm that this manuscript is an honest, accurate, and transparent account of the study being reported; that no important aspects of the study have been omitted; and that any discrepancies from the study as planned (and, if relevant, registered) have been explained.

## Supporting information


**Figure S1:** MR association between UC and lung functions.
**Figure S2:** MR association between UC and lung diseases.
**Figure S3:** MR association between CD and lung functions.
**Figure S4:** MR association between CD and lung diseases.
**Figure S5:** Leave‐one‐out sensitivity analyses.
**Figure S6:** Leave‐one‐out sensitivity analyses.
**Table S1:** The removed SNPs related to asthma, FEV1, FVC, PEF and tobacco smoking.
**Table S2:** SNPs as instrumental variables for UC and CD.

## Data Availability

The data that support the findings of this study are available from the corresponding author upon reasonable request.
