# Supplementary material for: Causal Effects of Inflammatory Bowel Disease on Lung Function and Disease: A Two‐Sample Mendelian Randomization Study
Source: Health Sci Rep. 2025 Jul 27;8(8):e71107. doi: 10.1002/hsr2.71107 (PMC12301568; doi:10.1002/hsr2.71107)
Supplement: Supplementary file 1 — Figure S1: MR association between UC and lung functions. Figure S2: MR association between UC and lung diseases. Figure S3: MR association between CD and lung functions. Figure S4: MR association between CD and lung diseases. Figure S5: Leave‐one‐out sensitivity analyses. Figure S6: Leave‐one‐out sensitivity analyses. Table S1: The removed SNPs related to asthma, FEV1, FVC, PEF and tobacco smoking. Table S2: SNPs as instrumental variables for UC and CD. [file HSR2-8-e71107-s001.docx]

**Supplementary material**


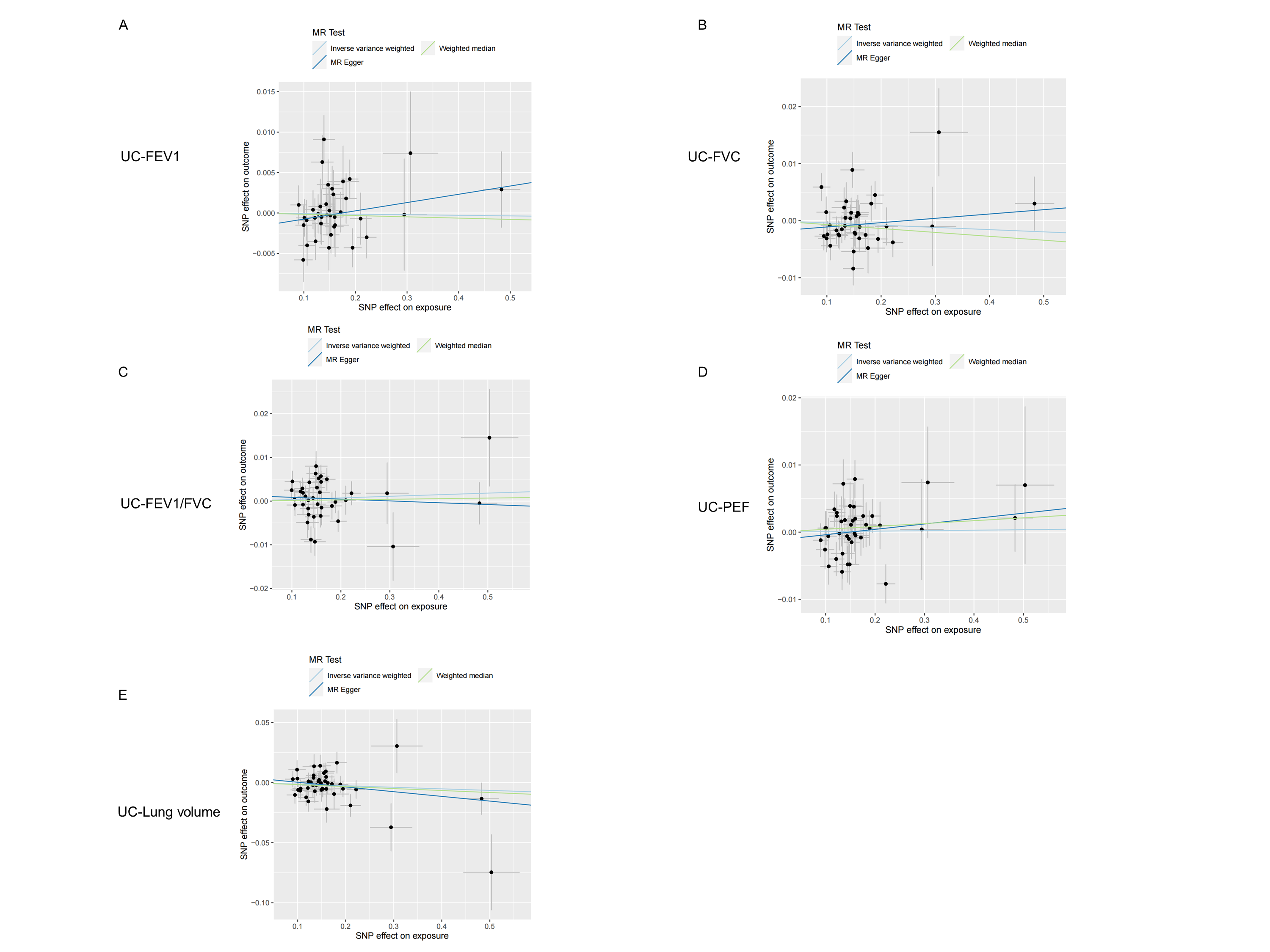


**Figure S1. MR association between UC and lung functions.** (A) Scatter plot for genetically predicted UC on FEV1; (B) Scatter plot for genetically predicted UC on FVC; (C) Scatter plot for genetically predicted UC on FEV1/FVC; (D) Scatter plot for genetically predicted UC on PEF; (E) Scatter plot for genetically predicted UC on Lung volume.


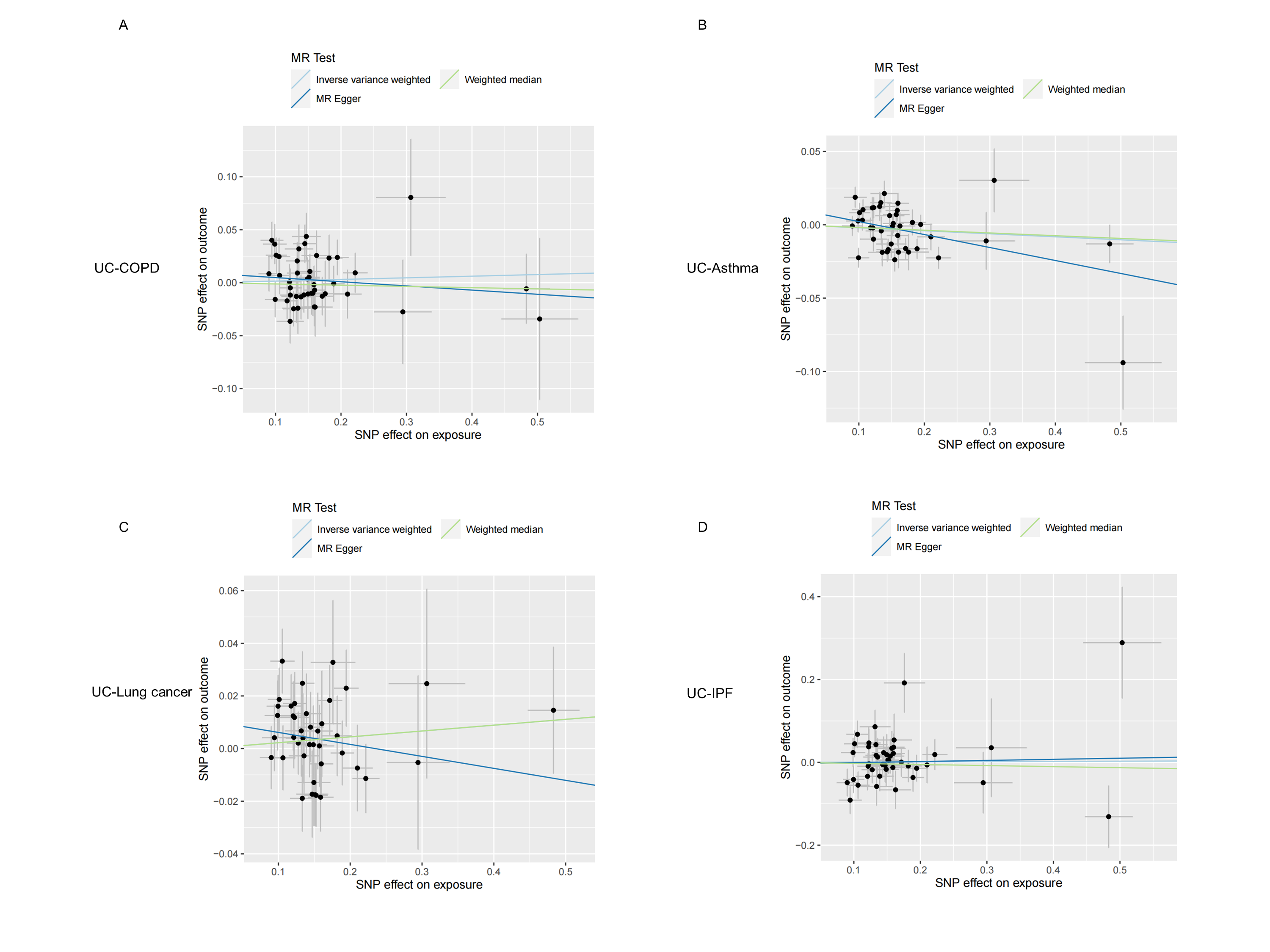


**Figure S2. MR association between UC and lung diseases.** (A) Scatter plot for genetically predicted UC on COPD; (B) Scatter plot for genetically predicted UC on Asthma; (C) Scatter plot for genetically predicted UC on Lung cancer; (D) Scatter plot for genetically predicted UC on IPF.


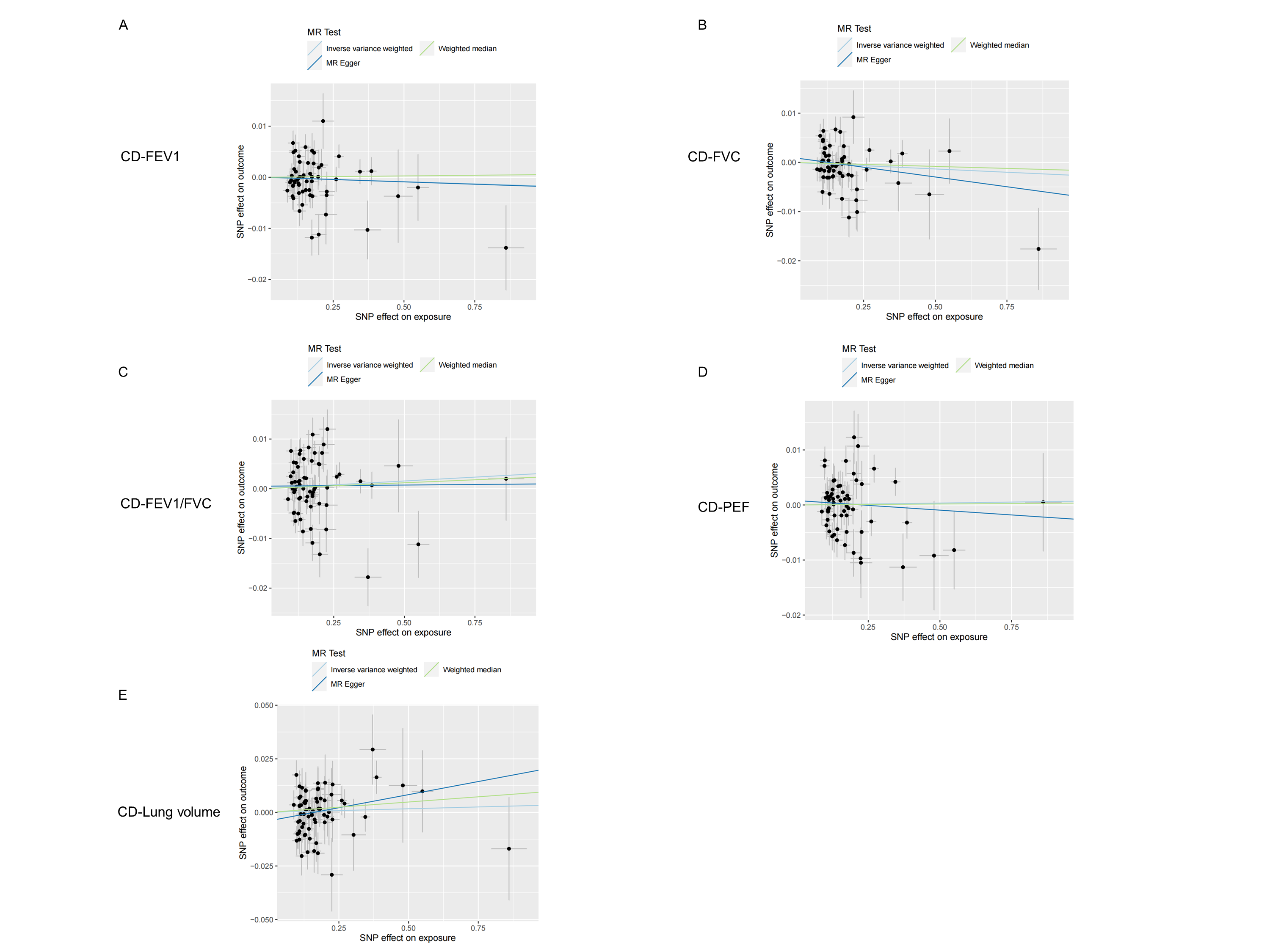


**Figure S3. MR association between CD and lung functions.** (A) Scatter plot for genetically predicted CD on FEV1; (B) Scatter plot for genetically predicted CD on FVC; (C) Scatter plot for genetically predicted CD on FEV1/FVC; (D) Scatter plot for genetically predicted CD on PEF; (E) Scatter plot for genetically predicted CD on Lung volume.


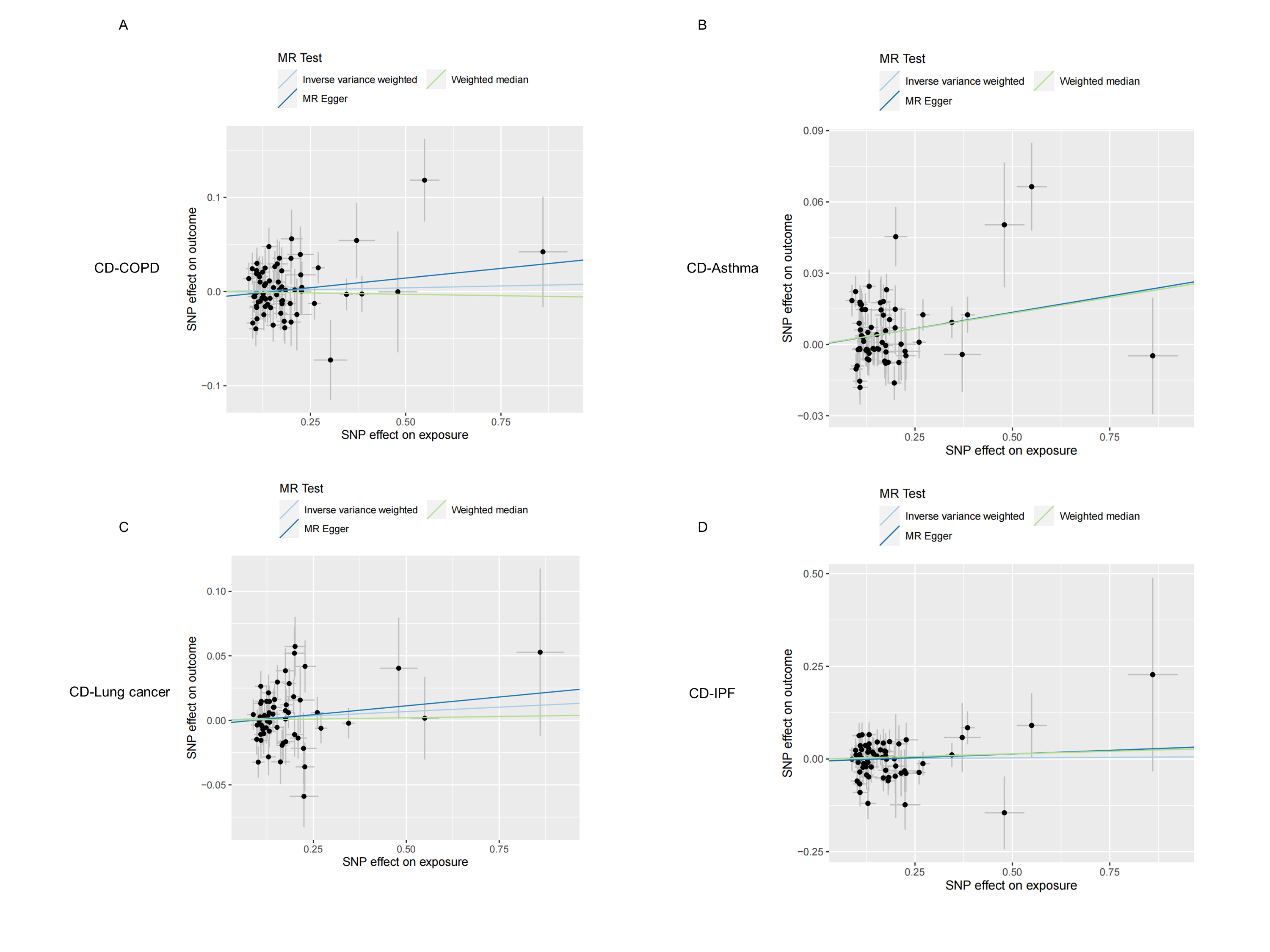


**Figure S4. MR association between CD and lung diseases.** (A) Scatter plot for genetically predicted CD on COPD; (B) Scatter plot for genetically predicted CD on Asthma; (C) Scatter plot for genetically predicted CD on Lung cancer; (D) Scatter plot for genetically predicted CD on IPF.


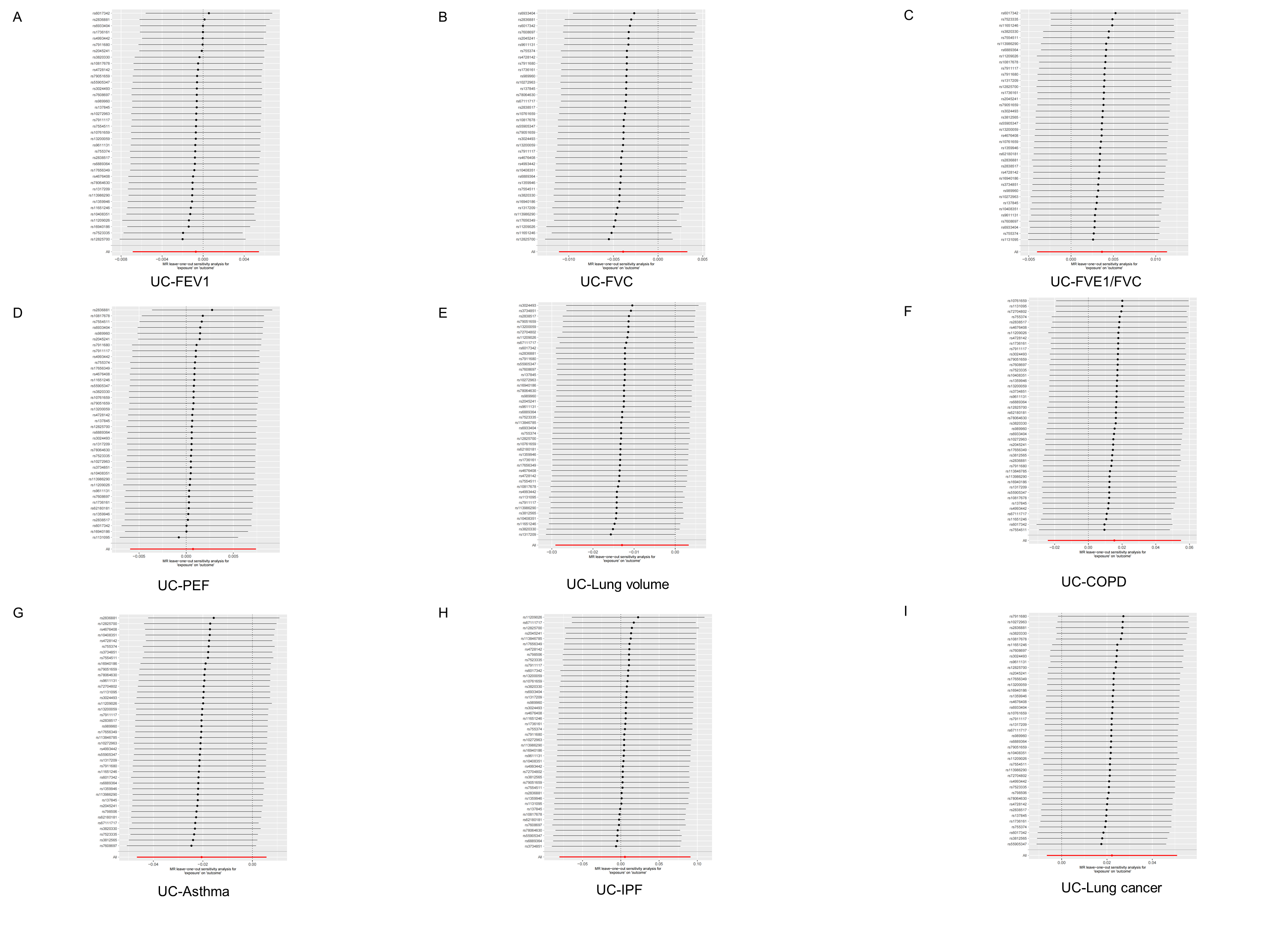


**Figure S5. Leave-one-out sensitivity analyses.** (A-E) MR leave-one-out sensitivity analysis for UC on Lung functions; (F-I) MR leave-one-out sensitivity analysis for UC on Lung diseases.


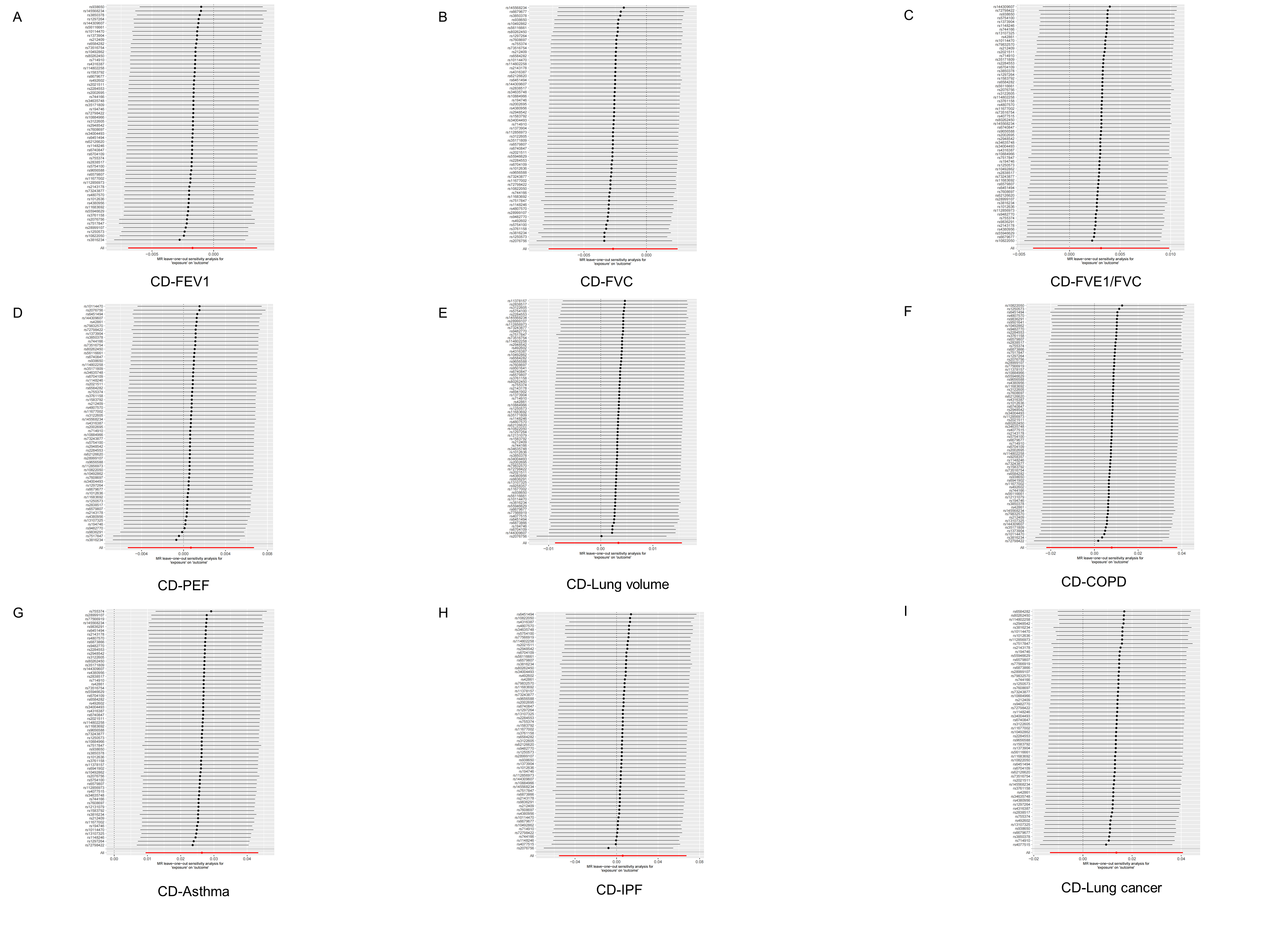


**Figure S6. Leave-one-out sensitivity analyses.** (A-E) MR leave-one-out sensitivity analysis for CD on Lung functions; (F-I) MR leave-one-out sensitivity analysis for CD on Lung diseases.

**Table S1.** The removed SNPs related to asthma, FEV1, FVC, PEF and tobacco smoking

| **Exposure** | | **SNP** | **Pleiotropy** |
| --- | --- | --- | --- |
| UC | rs17715902 | | Forced vital capacity, best measure |
| UC | rs9260809 | | Peak expiratory flow |
| UC | rs9271176 | | Asthma;Forced expiratory volume in 1-second;Forced vital capacity |
| UC | rs28383224 | | Forced vital capacity; |
| UC | rs2212434 | | Asthma;Treatment with ventolin 100micrograms inhaler;Self-reported hayfever or allergic rhinitis;Wheeze or whistling in the chest in last year |
| UC | rs56062135 | | Asthma;Hayfever, allergic rhinitis or eczema;Wheeze or whistling in the chest in last year;Treatment with ventolin 100micrograms inhaler |
| UC | rs12936409 | | Asthma;Treatment with ventolin 100micrograms inhaler;Wheeze or whistling in the chest in last year;Self-reported bronchitis;Hayfever, allergic rhinitis or eczema |
| UC | rs6062496 | | Forced vital capacity;Current tobacco smoking |
| CD | rs4343432 | | Forced vital capacity;Forced expiratory volume in 1-second |
| CD | rs6808936 | | Forced expiratory volume in 1-second;Forced vital capacity;Hayfever, allergic rhinitis or eczema |
| CD | rs62324212 | | Hayfever, allergic rhinitis or eczema;Asthma |
| CD | rs2188962 | | Asthma;Hayfever, allergic rhinitis or eczema |
| CD | rs181826 | | Asthma |
| CD | rs1321859 | | Asthma;Hayfever, allergic rhinitis or eczema;Treatment with ventolin 100micrograms inhaler |
| CD | rs111281598 | | Asthma |
| CD | rs61839660 | | Hayfever, allergic rhinitis or eczema;Allergic disease asthma hay fever or eczema; |
| CD | rs11236797 | | Hayfever, allergic rhinitis or eczema;Asthma;Wheeze or whistling in the chest in last year;Treatment with ventolin 100micrograms inhaler |
| CD | rs72743461 | | Asthma;Hayfever, allergic rhinitis or eczema;Wheeze or whistling in the chest in last year;Treatment with ventolin 100micrograms inhaler |
| CD | rs12936409 | | Asthma;Treatment with ventolin 100micrograms inhaler;Wheeze or whistling in the chest in last year;Self-reported bronchitis;Hayfever, allergic rhinitis or eczema |
| CD | rs6062496 | | Forced vital capacity;Current tobacco smoking |

**Table S2.** SNPs as instrumental variables for UC and CD

| Exposure | SNP | effect_allele.  exposure | other_allele.  exposure | se.  exposure | beta.  exposure | chr.  exposure | pos.  exposure | pval.  exposure | samplesize.  exposure | F-statistic |  |
| --- | --- | --- | --- | --- | --- | --- | --- | --- | --- | --- | --- |
| UC | rs7544646 | G | C | 0.016 | -0.1168 | 1 | 2496649 | 2.53E-13 | 45975 | 53.29 |  |
| UC | rs3024493 | A | C | 0.0209 | 0.21 | 1 | 206943968 | 7.46E-24 | 45975 | 100.9592271 |  |
| UC | rs3820330 | A | C | 0.0178 | -0.1587 | 1 | 20142413 | 3.91E-19 | 45975 | 79.49024744 |  |
| UC | rs11209026 | A | G | 0.0358 | -0.483 | 1 | 67705958 | 1.99E-41 | 45975 | 182.0238132 |  |
| UC | rs6658353 | C | G | 0.016 | -0.1569 | 1 | 161469054 | 1.17E-22 | 45975 | 96.16253906 |  |
| UC | rs7554511 | A | C | 0.0178 | -0.1448 | 1 | 200877562 | 4.27E-16 | 45975 | 66.17548289 |  |
| UC | rs7523335 | A | G | 0.021 | -0.1389 | 1 | 8180210 | 3.42E-11 | 45975 | 43.74877551 |  |
| UC | rs2816954 | A | T | 0.0229 | 0.1375 | 1 | 200105746 | 1.80E-09 | 45975 | 36.05242082 |  |
| UC | rs1317209 | A | G | 0.0203 | 0.1818 | 1 | 20140036 | 2.90E-19 | 45975 | 80.20393603 |  |
| UC | rs79051659 | A | G | 0.0264 | 0.1605 | 1 | 151757843 | 1.30E-09 | 45975 | 36.96087293 |  |
| UC | rs4654925 | C | G | 0.0159 | -0.2217 | 1 | 20227723 | 2.61E-44 | 45975 | 194.4182983 |  |
| UC | rs7608697 | C | A | 0.0161 | 0.1597 | 2 | 61204641 | 3.03E-23 | 45975 | 98.39161298 |  |
| UC | rs55905347 | A | G | 0.0166 | 0.1054 | 2 | 199638507 | 2.09E-10 | 45975 | 40.31484976 |  |
| UC | rs62180181 | T | C | 0.0171 | 0.1226 | 2 | 199852010 | 8.08E-13 | 45975 | 51.40302999 |  |
| UC | rs4676408 | A | G | 0.0167 | 0.1433 | 2 | 241574401 | 1.19E-17 | 45975 | 73.63078633 |  |
| UC | rs1811711 | G | C | 0.0223 | -0.1299 | 2 | 228670476 | 6.09E-09 | 45975 | 33.93193107 |  |
| UC | rs1131095 | C | T | 0.0168 | 0.1593 | 3 | 49714225 | 2.18E-21 | 45975 | 89.91103316 |  |
| UC | rs755374 | T | C | 0.0171 | 0.1714 | 5 | 158829294 | 9.73E-24 | 45975 | 100.4683834 |  |
| UC | rs72704802 | T | C | 0.0206 | -0.1223 | 5 | 554211 | 2.89E-09 | 45975 | 35.24670091 |  |
| UC | rs6889364 | A | G | 0.0228 | 0.1318 | 5 | 40347469 | 7.87E-09 | 45975 | 33.41651277 |  |
| UC | rs17656349 | T | C | 0.0159 | 0.09 | 5 | 149605994 | 1.54E-08 | 45975 | 32.03987184 |  |
| UC | rs67111717 | G | A | 0.0171 | 0.0944 | 5 | 176790162 | 3.27E-08 | 45975 | 30.47556513 |  |
| UC | rs9267798 | C | G | 0.028 | 0.2486 | 6 | 32044834 | 6.54E-19 | 45975 | 78.82903061 |  |
| UC | rs3734851 | A | G | 0.0584 | 0.5033 | 6 | 31030303 | 6.58E-18 | 45975 | 74.27252123 |  |
| UC | rs13200059 | A | G | 0.0436 | 0.2944 | 6 | 111943234 | 1.48E-11 | 45975 | 45.59346856 |  |
| UC | rs6933404 | C | T | 0.0188 | 0.1486 | 6 | 137959235 | 2.69E-15 | 45975 | 62.47725215 |  |
| UC | rs113986290 | T | C | 0.0531 | -0.3066 | 6 | 19781009 | 7.59E-09 | 45975 | 33.33920649 |  |
| UC | rs798506 | C | T | 0.0179 | -0.1206 | 7 | 2788912 | 1.47E-11 | 45975 | 45.39296526 |  |
| UC | rs4728142 | A | G | 0.0158 | 0.0995 | 7 | 128573967 | 3.23E-10 | 45975 | 39.65810768 |  |
| UC | rs989960 | T | C | 0.016 | -0.1214 | 7 | 107445727 | 3.28E-14 | 45975 | 57.57015625 |  |
| UC | rs10272963 | T | C | 0.016 | -0.1512 | 7 | 107486902 | 4.11E-21 | 45975 | 89.3025 |  |
| UC | rs1887428 | C | G | 0.0166 | -0.167 | 9 | 4984530 | 9.65E-24 | 45975 | 101.2084483 |  |
| UC | rs10817678 | A | G | 0.017 | 0.1332 | 9 | 117579457 | 4.42E-15 | 45975 | 61.39183391 |  |
| UC | rs3812565 | C | T | 0.016 | 0.1335 | 9 | 139272502 | 6.50E-17 | 45975 | 69.61816406 |  |
| UC | rs10761659 | G | A | 0.016 | 0.1276 | 10 | 64445564 | 1.33E-15 | 45975 | 63.600625 |  |
| UC | rs7911117 | G | T | 0.0239 | -0.1342 | 10 | 27179596 | 1.84E-08 | 45975 | 31.52892982 |  |
| UC | rs7911680 | C | A | 0.0159 | -0.1525 | 10 | 101293468 | 6.71E-22 | 45975 | 91.99102092 |  |
| UC | rs2045241 | A | G | 0.0169 | -0.1063 | 11 | 114428783 | 2.83E-10 | 45975 | 39.56335562 |  |
| UC | rs12825700 | A | G | 0.0161 | 0.1889 | 12 | 68492980 | 7.33E-32 | 45975 | 137.6613942 |  |
| UC | rs1359946 | A | G | 0.0202 | 0.1571 | 13 | 27536972 | 6.58E-15 | 45975 | 60.48527105 |  |
| UC | rs11645239 | G | C | 0.02 | -0.1174 | 16 | 23847062 | 4.14E-09 | 45975 | 34.4569 |  |
| UC | rs7203363 | A | T | 0.0189 | 0.1071 | 16 | 68587692 | 1.41E-08 | 45975 | 32.11111111 |  |
| UC | rs16940186 | C | T | 0.0214 | 0.1357 | 16 | 86009740 | 2.18E-10 | 45975 | 40.20982182 |  |
| UC | rs113846785 | CG | C | 0.0229 | -0.1627 | 17 | 70611194 | 1.15E-12 | 45975 | 50.47823268 |  |
| UC | rs11651246 | G | T | 0.0219 | 0.147 | 17 | 40759937 | 2.01E-11 | 45975 | 45.05535748 |  |
| UC | rs10408351 | A | G | 0.0204 | 0.1548 | 19 | 33754044 | 2.92E-14 | 45975 | 57.58131488 |  |
| UC | rs78064630 | A | G | 0.0308 | 0.1759 | 19 | 10562802 | 1.08E-08 | 45975 | 32.61596601 |  |
| UC | rs6017342 | C | A | 0.017 | 0.1944 | 20 | 43065028 | 3.95E-30 | 45975 | 130.7659516 |  |
| UC | rs2836881 | T | G | 0.0186 | -0.2217 | 21 | 40466299 | 1.11E-32 | 45975 | 142.0710198 |  |
| UC | rs2838517 | C | T | 0.016 | -0.1177 | 21 | 45613825 | 1.78E-13 | 45975 | 54.11441406 |  |
| UC | rs1736161 | A | G | 0.0161 | -0.1227 | 21 | 16833222 | 2.22E-14 | 45975 | 58.08143976 |  |
| UC | rs9611131 | C | T | 0.0227 | -0.1494 | 22 | 39662480 | 5.11E-11 | 45975 | 43.31611326 |  |
| UC | rs4993442 | T | G | 0.0179 | -0.0988 | 22 | 30253256 | 3.54E-08 | 45975 | 30.46546612 |  |
| UC | rs137845 | G | A | 0.0158 | 0.1011 | 22 | 50439430 | 1.50E-10 | 45975 | 40.94379907 |  |
| CD | rs12131079 | T | C | 0.0174 | -0.1088 | 1 | 155319668 | 3.99E-10 | 40266 | 39.0984278 |  |
| CD | rs35730213 | C | G | 0.0181 | -0.1166 | 1 | 200874229 | 1.17E-10 | 40266 | 41.49922164 |  |
| CD | rs3122605 | A | G | 0.0227 | -0.1748 | 1 | 206955041 | 1.24E-14 | 40266 | 59.29678434 |  |
| CD | rs114802258 | T | C | 0.0384 | -0.2245 | 1 | 160831855 | 5.11E-09 | 40266 | 34.17985704 |  |
| CD | rs4316387 | C | T | 0.0189 | -0.1292 | 1 | 197595569 | 7.74E-12 | 40266 | 46.73060665 |  |
| CD | rs6679677 | A | C | 0.0286 | -0.2275 | 1 | 114303808 | 1.77E-15 | 40266 | 63.27479339 |  |
| CD | rs6704109 | T | C | 0.0181 | 0.1748 | 1 | 172857050 | 5.10E-22 | 40266 | 93.26650591 |  |
| CD | rs7517847 | G | T | 0.0165 | -0.3447 | 1 | 67681669 | 5.84E-97 | 40266 | 436.4300826 |  |
| CD | rs11378157 | AG | A | 0.0185 | -0.1376 | 2 | 103065522 | 9.29E-14 | 40266 | 55.3214317 |  |
| CD | rs11683692 | C | T | 0.038 | -0.2144 | 2 | 145509615 | 1.75E-08 | 40266 | 31.8333518 |  |
| CD | rs11677002 | C | T | 0.0163 | -0.1124 | 2 | 28614401 | 4.57E-12 | 40266 | 47.55075464 |  |
| CD | rs34004493 | G | A | 0.0179 | 0.1258 | 2 | 231154012 | 2.00E-12 | 40266 | 49.3918417 |  |
| CD | rs3816234 | A | G | 0.0162 | 0.2704 | 2 | 234185999 | 1.51E-62 | 40266 | 278.6014327 |  |
| CD | rs55946629 | A | C | 0.0231 | 0.1755 | 2 | 43851246 | 2.85E-14 | 40266 | 57.72052623 |  |
| CD | rs7608697 | C | A | 0.0163 | 0.1229 | 2 | 61204641 | 4.03E-14 | 40266 | 56.84974971 |  |
| CD | rs6740847 | G | A | 0.0161 | -0.104 | 2 | 182308352 | 9.72E-11 | 40266 | 41.72678523 |  |
| CD | rs1583792 | T | C | 0.016 | -0.0882 | 2 | 198900288 | 3.26E-08 | 40266 | 30.38765625 |  |
| CD | rs56116661 | T | C | 0.0212 | -0.1312 | 3 | 188401160 | 5.67E-10 | 40266 | 38.2997508 |  |
| CD | rs9836291 | A | G | 0.017 | 0.1722 | 3 | 49697459 | 3.77E-24 | 40266 | 102.6049827 |  |
| CD | rs2581828 | G | C | 0.0162 | -0.0941 | 3 | 53133149 | 6.46E-09 | 40266 | 33.7403216 |  |
| CD | rs73243877 | G | A | 0.0212 | 0.1164 | 4 | 26047616 | 4.12E-08 | 40266 | 30.14631541 |  |
| CD | rs13107325 | T | C | 0.0284 | 0.2006 | 4 | 103188709 | 1.66E-12 | 40266 | 49.891341 |  |
| CD | rs6579807 | T | C | 0.0244 | 0.1993 | 5 | 150286845 | 3.44E-16 | 40266 | 66.71675961 |  |
| CD | rs755374 | T | C | 0.0174 | 0.1969 | 5 | 158829294 | 1.38E-29 | 40266 | 128.0539371 |  |
| CD | rs6451494 | C | T | 0.0166 | 0.2605 | 5 | 40411291 | 8.26E-56 | 40266 | 246.2630643 |  |
| CD | rs112856973 | C | T | 0.0243 | -0.1612 | 5 | 40530242 | 3.61E-11 | 40266 | 44.00657081 |  |
| CD | rs6873866 | C | T | 0.0164 | -0.1314 | 5 | 96247810 | 1.35E-15 | 40266 | 64.19527067 |  |
| CD | rs1012636 | T | G | 0.0198 | 0.1291 | 6 | 20674811 | 7.01E-11 | 40266 | 42.51303438 |  |
| CD | rs73516754 | C | A | 0.0169 | 0.1423 | 6 | 106459738 | 4.04E-17 | 40266 | 70.89839291 |  |
| CD | rs35171809 | G | A | 0.0159 | 0.1566 | 6 | 167432766 | 9.07E-23 | 40266 | 97.00391598 |  |
| CD | rs6941902 | C | T | 0.0273 | 0.163 | 6 | 32684630 | 2.39E-09 | 40266 | 35.64921037 |  |
| CD | rs7753014 | G | C | 0.0163 | -0.0989 | 6 | 21441035 | 1.39E-09 | 40266 | 36.81437013 |  |
| CD | rs145568234 | G | T | 0.0633 | 0.8602 | 6 | 32247045 | 4.31E-42 | 40266 | 184.6679195 |  |
| CD | rs9482770 | C | T | 0.0162 | 0.0987 | 6 | 127443092 | 1.01E-09 | 40266 | 37.1196845 |  |
| CD | rs9501641 | T | C | 0.0432 | 0.3027 | 6 | 32450319 | 2.57E-12 | 40266 | 49.09727045 |  |
| CD | rs9258357 | C | T | 0.0216 | -0.1179 | 6 | 29751129 | 5.00E-08 | 40266 | 29.79340278 |  |
| CD | rs212409 | A | G | 0.0162 | -0.1096 | 6 | 159470058 | 1.49E-11 | 40266 | 45.77107148 |  |
| CD | rs9656588 | C | T | 0.0173 | 0.1183 | 7 | 50306780 | 8.73E-12 | 40266 | 46.76029938 |  |
| CD | rs938650 | A | G | 0.0247 | -0.1747 | 8 | 129552540 | 1.65E-12 | 40266 | 50.02555361 |  |
| CD | rs4380956 | A | G | 0.0165 | 0.132 | 8 | 126529074 | 1.15E-15 | 40266 | 64 |  |
| CD | rs79832570 | C | T | 0.0344 | 0.2234 | 8 | 145097720 | 8.90E-11 | 40266 | 42.17445241 |  |
| CD | rs10114470 | C | T | 0.0177 | 0.1687 | 9 | 117547772 | 1.76E-21 | 40266 | 90.84136104 |  |
| CD | rs1887428 | C | G | 0.0169 | -0.166 | 9 | 4984530 | 8.54E-23 | 40266 | 96.48121564 |  |
| CD | rs4077515 | T | C | 0.0162 | 0.1848 | 9 | 139266496 | 3.14E-30 | 40266 | 130.1289438 |  |
| CD | rs10884966 | A | G | 0.0171 | 0.1131 | 10 | 112185596 | 4.13E-11 | 40266 | 43.74546014 |  |
| CD | rs2002695 | G | A | 0.0189 | -0.1293 | 10 | 30805480 | 8.31E-12 | 40266 | 46.80297304 |  |
| CD | rs10822050 | C | T | 0.0162 | 0.1827 | 10 | 64438771 | 2.35E-29 | 40266 | 127.1882716 |  |
| CD | rs2675670 | C | G | 0.0161 | 0.1074 | 10 | 75655628 | 2.89E-11 | 40266 | 44.49967208 |  |
| CD | rs1148246 | T | C | 0.0167 | -0.1323 | 10 | 35496626 | 2.09E-15 | 40266 | 62.76055075 |  |
| CD | rs1250573 | A | G | 0.0179 | -0.1522 | 10 | 81042475 | 1.92E-17 | 40266 | 72.29749384 |  |
| CD | rs6584282 | G | A | 0.016 | -0.1658 | 10 | 101286495 | 3.44E-25 | 40266 | 107.3814063 |  |
| CD | rs28999107 | T | G | 0.0178 | 0.1083 | 12 | 6493100 | 1.06E-09 | 40266 | 37.01833733 |  |
| CD | rs77566919 | A | G | 0.0185 | -0.1089 | 12 | 113163656 | 4.13E-09 | 40266 | 34.65072316 |  |
| CD | rs34635748 | T | C | 0.0504 | 0.4794 | 12 | 40824663 | 1.95E-21 | 40266 | 90.4763322 |  |
| CD | rs1373904 | G | A | 0.0189 | 0.141 | 13 | 44475398 | 9.11E-14 | 40266 | 55.65633661 |  |
| CD | rs194746 | T | C | 0.0161 | 0.0975 | 14 | 69282887 | 1.24E-09 | 40266 | 36.67393233 |  |
| CD | rs3850378 | C | T | 0.0267 | 0.199 | 14 | 88417517 | 8.31E-14 | 40266 | 55.54994459 |  |
| CD | rs2021511 | T | C | 0.0182 | -0.1082 | 16 | 11344903 | 2.63E-09 | 40266 | 35.3436783 |  |
| CD | rs42861 | G | A | 0.0167 | 0.1243 | 16 | 28494421 | 8.87E-14 | 40266 | 55.39994263 |  |
| CD | rs2076756 | G | A | 0.0174 | 0.385 | 16 | 50756881 | 1.80E-108 | 40266 | 489.5792047 |  |
| CD | rs7195228 | G | C | 0.0209 | -0.1327 | 16 | 50353529 | 2.09E-10 | 40266 | 40.31338568 |  |
| CD | rs72798422 | C | T | 0.0382 | 0.5495 | 16 | 50866917 | 6.05E-47 | 40266 | 206.922953 |  |
| CD | rs10492862 | A | C | 0.0176 | 0.1067 | 16 | 82867456 | 1.26E-09 | 40266 | 36.75390625 |  |
| CD | rs2948542 | G | A | 0.0163 | 0.1016 | 17 | 25856486 | 5.15E-10 | 40266 | 38.85189507 |  |
| CD | rs714910 | C | A | 0.0181 | -0.1531 | 17 | 32617265 | 2.49E-17 | 40266 | 71.54729709 |  |
| CD | rs744166 | G | A | 0.0162 | -0.1142 | 17 | 40514201 | 1.80E-12 | 40266 | 49.69379668 |  |
| CD | rs80262450 | A | G | 0.0244 | 0.2268 | 18 | 12818922 | 1.34E-20 | 40266 | 86.39854878 |  |
| CD | rs144309607 | T | C | 0.047 | -0.3712 | 19 | 10492274 | 2.69E-15 | 40266 | 62.37638751 |  |
| CD | rs62126620 | A | G | 0.0201 | 0.144 | 19 | 33753200 | 8.61E-13 | 40266 | 51.32546224 |  |
| CD | rs4807570 | A | G | 0.0193 | 0.1811 | 19 | 1123652 | 6.03E-21 | 40266 | 88.04856506 |  |
| CD | rs492602 | G | A | 0.0162 | 0.1084 | 19 | 49206417 | 2.33E-11 | 40266 | 44.77427221 |  |
| CD | rs3761158 | A | G | 0.0165 | -0.1098 | 20 | 44634912 | 2.65E-11 | 40266 | 44.28297521 |  |
| CD | rs1297264 | G | A | 0.0163 | -0.1769 | 21 | 16816017 | 1.59E-27 | 40266 | 117.7824156 |  |
| CD | rs2284553 | G | A | 0.0165 | 0.1277 | 21 | 34776695 | 1.14E-14 | 40266 | 59.89821855 |  |
| CD | rs2838517 | C | T | 0.0162 | -0.1456 | 21 | 45613825 | 2.03E-19 | 40266 | 80.77793019 |  |
| CD | rs2143178 | C | T | 0.0223 | -0.2087 | 22 | 39660829 | 6.84E-21 | 40266 | 87.58609664 |  |
| CD | rs5754100 | C | T | 0.0206 | 0.1687 | 22 | 21916166 | 3.02E-16 | 40266 | 67.06496842 |  |
